# In pursuit of degenerative brain disease diagnosis: Dementia biomarkers detected by DNA aptamer-attached portable graphene biosensor

**DOI:** 10.1073/pnas.2311565120

**Published:** 2023-11-13

**Authors:** Tyler Andrew Bodily, Anirudh Ramanathan, Shanhong Wei, Abhijith Karkisaval, Nemil Bhatt, Cynthia Jerez, Md Anzarul Haque, Armando Ramil, Prachi Heda, Yi Wang, Sanjeev Kumar, Mikayla Leite, Tie Li, Jianlong Zhao, Ratnesh Lal

**Affiliations:** ^a^Department of Bioengineering, University of California, San Diego, CA 92093; ^b^State Key Laboratory of Transducer Technology, Shanghai Institute of Microsystem and Information Technology, Chinese Academy of Sciences, Shanghai 200050, China; ^c^University of Chinese Academy of Sciences, Beijing 100049, China; ^d^Department of Mechanical and Aerospace Engineering, University of California, San Diego, CA 92093; ^e^Mitchell Center for Neurodegenerative Disorders, Department of Neurology, University of Texas Medical Branch, Galveston, TX 77555; ^f^Department of Computer Science, University of Illinois Urbana-Champaign, Champaign, IL 61820; ^g^Materials Science and Engineering Program, University of California, San Diego, CA 92093

**Keywords:** aptamer, graphene, biosensor, dementia, Alzheimer’s disease

## Abstract

Dementia is a brain disease which results in irreversible and progressive loss of cognition and motor activity. Despite global efforts, there is no simple and reliable diagnosis or treatment option. Current diagnosis involves indirect testing of commonly inaccessible biofluids and low-resolution brain imaging. We have developed a portable, wireless readout-based Graphene field-effect transistor (GFET) biosensor platform that can detect viruses, proteins, and small molecules with single-molecule sensitivity and specificity. We report the detection of three important amyloids, namely, Amyloid beta (Aβ), Tau (τ), and α-Synuclein (αS) using DNA aptamer nanoprobes. These amyloids were isolated, purified, and characterized from the autopsied brain tissues of Alzheimer’s Disease (AD) and Parkinson’s Disease (PD) patients. The limit of detection (LoD) of the sensor is 10 fM, 1–10 pM, 10–100 fM for Aβ, τ, and αS, respectively. Synthetic as well as autopsied brain-derived amyloids showed a statistically significant sensor response with respect to derived thresholds, confirming the ability to define diseased vs. nondiseased states. The detection of each amyloid was specific to their aptamers; Aβ, τ, and αS peptides when tested, respectively, with aptamers nonspecific to them showed statistically insignificant cross-reactivity. Thus, the aptamer-based GFET biosensor has high sensitivity and precision across a range of epidemiologically significant AD and PD variants. This portable diagnostic system would allow at-home and POC testing for neurodegenerative diseases globally.

## Significance

Our memories define us and connect us to others. Without them, we are lost. This is the driving force behind the global push to treat neurodegenerative diseases of the older population. How does one know they have a disease that has few outward symptoms until later stages? The current testing methods for diseases such as Alzheimer’s and Parkinson’s require a spinal tap and imaging tests such as MRI. This has made early detection of these diseases an incredible challenge. This work highlights a DNA aptamer-modified graphene field-effect transistor biosensor to detect unprocessed biomarker proteins in easily accessible biofluids derived from patients with Alzheimer’s disease, in pursuit of an affordable early-onset detection of neurodegenerative diseases.

One of the greatest modern challenges is an effective prevention and treatment of degenerative brain disorders such as Alzheimer’s (AD) and Parkinson’s disease (PD). Individuals with AD and PD begin to lose cognitive and motor faculties and suffer from severe and progressive dementia until their death (1–3). In addition to the debilitating impact on the quality of life of patients with dementia, the families, friends, and caregivers also experience often unbearable hardship. People today are living longer, and the significant aging population has brought to the forefront the evolving endemic of neurodegenerative diseases. It is estimated that by the year 2060, there will be 14 million Americans alone afflicted with AD (2). Other neurodegenerative diseases such as PD are also appearing at an increasing rate (4). Though there has been a concerted effort to understand, diagnose, treat, and cure neurodegenerative diseases, the progress for early and simple diagnosis is abysmal.

The progression of neurodegenerative diseases, especially AD, is historically associated with Amyloid-β (Aβ) protein plaque formation within the extracellular space and Tau neurofibrillary tangles inside neurons of the brain. The two major isoforms Aβ_1−40_ and Aβ_1−42_ are formed via the successive proteolytic cleavage of amyloid precursor protein (APP). Due to their high aggregation propensity, Aβ proteins oligomerize and eventually form insoluble amyloid fibrils present in the core of senile plaques, characteristic of AD (5). The current prevailing view in the AD community is that the soluble Aβ oligomers are connected to early AD symptoms and disease onset (5, 6). Upon hyperphosphorylation, microtubule-associated protein Tau (τ) can form helical filaments called the neurofibrillary tangles (NFTs). These NFTs follow a characteristic spatiotemporal progression in AD-diagnosed individuals (5). As the NFTs and plaque concentrations grow, there is an increase in neurite cell death and eventual decline in cognitive ability and death (7). Together NFTs and Aβ plaques form the core of AD pathophysiology and progression.

PD is identified by a distinct α-Synuclein (αS)-linked pathophysiology and histological hallmarks, specifically, the presence of Lewy bodies (LBs) that occur in dopaminergic neurons of the substantia nigra pars compacta (SNpc) neurons (8). The death of the dopaminergic neurons has been linked to loss of autonomic, motor control, and cognitive ability leading to dementia. Recent work has shown that αS protein is the primary fibrillar component of LB and that αS overexpression can cause dopaminergic neuron cell death (9). The precise pathophysiology between αS and PD diagnosis is not clearly understood, but many studies point to some disruption of dopamine function (i.e., storage, efflux, interaction with SNARE complex) (9).

AD, PD, and other neurodegenerative diseases often have biological onset decades prior to any clinical diagnosis or identifiable traits (1, 4). Clinicians often use amnestic phenotypes, and visual/auditory or vocal impairment as key features of dementia caused by AD, but studies have indicated that many patients diagnosed with AD via autopsy never showed clinically diagnosable levels of impairment (1, 5). The depletion of soluble Aβ_1–42_ and the reduction in the ratio of Aβ_1–42_/Aβ_1–40_ levels in bodily fluids such as CSF have been shown to be reliable biomarkers in the diagnosis of AD. (5, 10). In the case of PD, the protein αS is involved in various stages of disease progression and is a promising biomarker for the diagnosis of PD since aggregates are closely correlated with PD pathogenesis (9, 11).

The early detection of PD and AD prior to the onset of phenotypic changes is critical for effective prevention and treatment. Although some commercial diagnostic tests are being marketed to test for AD and PD biomarkers from blood, they are mostly designed as a “sample collection” from the user but the actual test is run by qualified professionals with expensive analysis procedures and equipment (12, 13). The goal is early detection and enhanced longitudinal studies in the preclinical stages. Some promising approaches for a point-of-care (POC) test for rapid and accurate detection of Aβ, Tau, and αS concentration using CSF, brain, and blood have also been demonstrated (14–17). However, there is no POC or at-home testing of commonly accessible biofluids, such as blood, saliva, and urine containing Aβ, Tau, and αS with single-molecule sensitivity and specificity to correlate with predictive value.

**Fig. 1.**
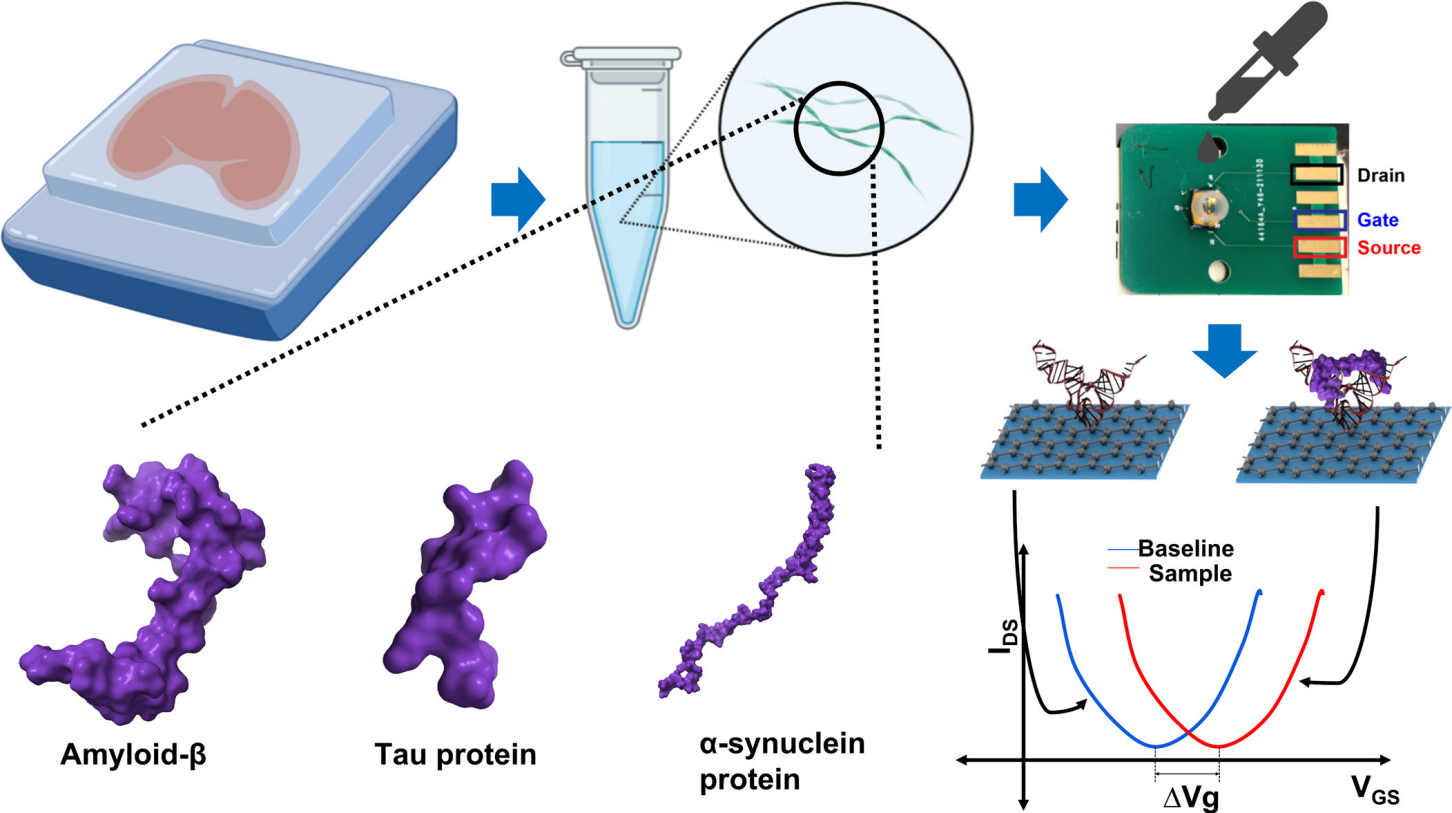
Schematic of sensing platform and detection methodology. A schematic of the biosensor testing process utilized throughout the paper. *Top-left*: Amyloid proteins were immunoprecipitated from homogenized brain tissue from autopsied AD and PD patients. The brain-derived proteins were then applied to the silicone well in the GFET biosensor chip (*Top-right*). *Bottom-left*: The 3D models of neurodegenerative amyloid proteins (PDB IDs for Aβ, Tau, and αS are 6cvj, 1xq8, and 2mxu) are shown produced with the software, ChimeraX (19–22). *Bottom-right*: The graphene surface of the GFET chip is functionalized with an aptamer (probe) that binds to the specific analyte (shown as Aβ monomer) and analyte-probe-specific interaction shifts the Dirac point in the plots of the gate voltage vs drain–source currents. The Dirac point shift between the baseline (control I–V curve without an analyte) and the I–V curve in the presence of the biomarker sample is recorded and analyzed by the reader (18).

Recently, we have developed a graphene field-effect transistor (GFET)-based biosensor platform for the detection of SARS-CoV2 and its variants (Fig. 1) (18) and detected as low as 5–7 live viruses per 10 μL and subfemtomolar concentrations of spike/nucleoproteins. The sensor consists of a single-atomic layer of graphene in between a source and drain electrode with a liquid-gated electrode for the generation of the field effect at the graphene surface. We adapted this platform for the detection of specific protein biomarkers for AD and PD. The graphene surface electric charge transfer was modulated with aptamer specific to Aβ, Tau, and αS. The amyloid–aptamer binding-induced change was detected as the shift in the Dirac point—the minimum value (i.e., charge neutrality point) in the I–V curve (18).

Briefly, we first characterized the functionality of the GFET platform using Raman spectroscopy, atomic force microscopy (AFM), and electrical measurements. We then functionalized the graphene surface with identified high-affinity aptamers (*SI Appendix*, Table S1) specific to various neurodegenerative disease-associated proteins, specifically, Aβ_1–42_, Tau441, and αS. We then quantified our aptasensor’s specificity and limit of detection (LoD) for these proteins using the synthetic isoforms of the proteins in controlled buffer environment. To develop a reliable sensor for amyloid protein biomarker detection in AD patient samples, we tested our biosensor against brain-derived amyloid proteins. Through appropriate control experiments, we demonstrate high specificity and low cross-reactivity. Overall, our results indicate that the aptamer-GFET sensor can specifically detect protein biomarkers for AD and PD with high fidelity.

## Results

### GFET Characterization.

We used 1-pyrene butanoic acid NHS ester (PBASE) as a linker between graphene and aptamer. The chemical functionalization of PBASE on graphene was examined by Raman Spectroscopy on the bare graphene surface and compared to a PBASE-modified graphene surface, using a 532-nm laser with 20x magnification (*SI Appendix*, Fig. S2 *C* and *D*). The map of the 2D/G peak intensity ratios on 96 consecutive points on the brightfield image with 20-μm pitch indicates an average ratio of 1.9 corresponding to a uniform graphene monolayer on our sensor (23) (*SI Appendix*, Fig. S2*A*). After PBASE conjugation, the ratio lowered to around 1.29 (*SI Appendix*, Fig. S2*B*), along with the formation of significant D and D′ peaks. This indicates pyrene group binding and enhanced sp^2^ bonding (24).

**Fig. 2.**
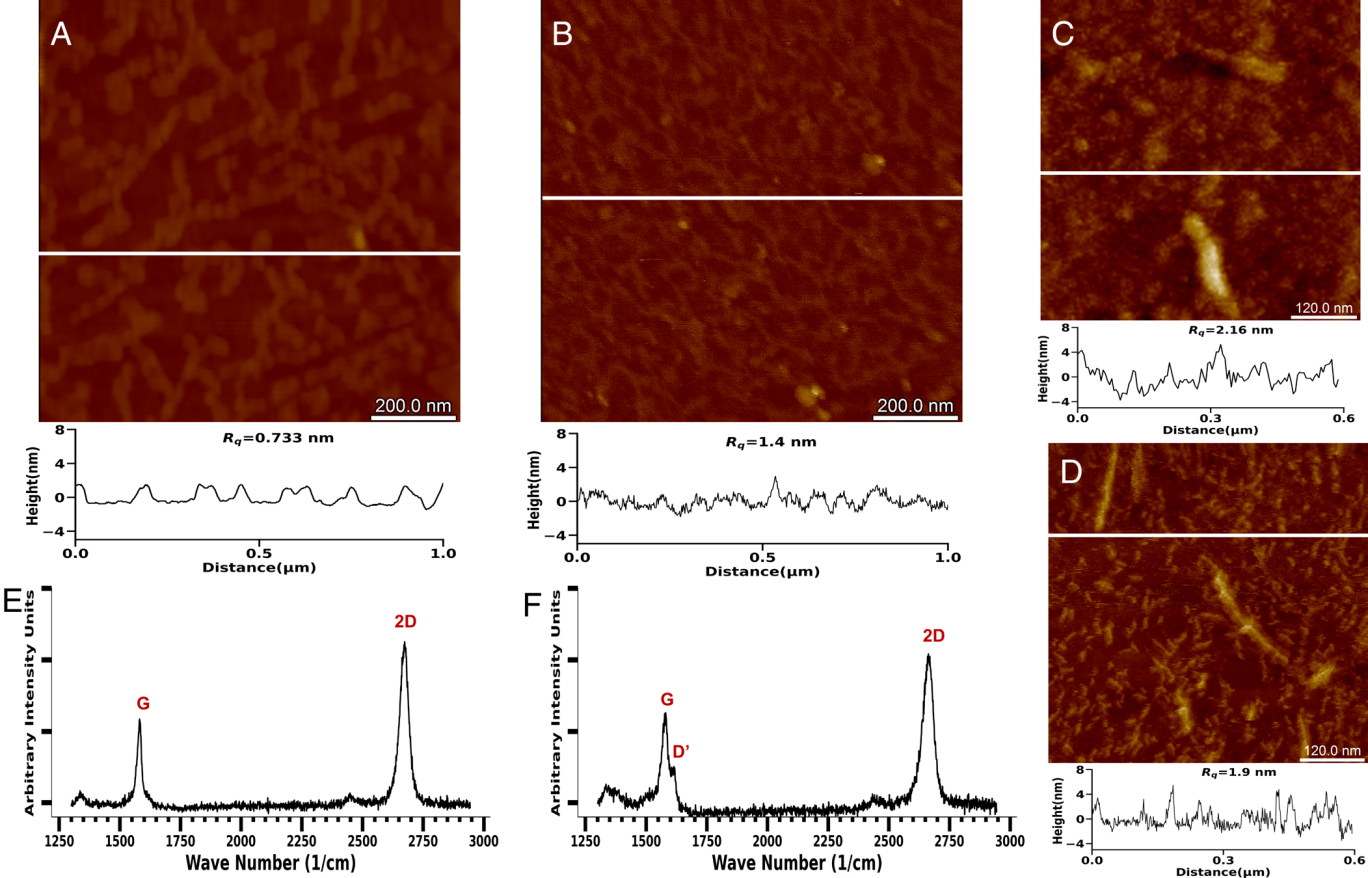
GFET Sensor Characterization via Raman Spectroscopy and AFM. (*A*) AFM height image of a bare GFET sensor with its section profile (denoted by the white line in *A*–*D*). (*B*) AFM height image of a PBASE functionalized sensor. (*C*) AFM height image of sensor post fully functionalizing and adding Aβ_1–42_. (*D*) AFM height image of Aβ_1–42_ on freshly cleaved mica showing a distribution of lower order oligomers and fibrils. R_q_ is the RMS roughness of the entire AFM image. All heights in AFM images are between 0 and 30 nm. (*E*) Raman spectroscopy plot of bare graphene chip on a single 20 μm × 20 μm area. (*F*) Raman spectroscopy plot of PBASE functionalized graphene on a single 20 μm × 20 μm area.

The morphology of the graphene surface was examined by AFM. The RMS surface roughness (R_q_) of bare graphene was 0.733 ± 0.20 nm. PBASE functionalization increased surface roughness to 1.4 ± 0.6 nm. The additional roughness after the PBASE addition phase indicates the successful binding of pyrene and graphene pi–pi stacking interaction. An increase in the roughness was also observed when Aβ_1–42_ was added to the fully functionalized chip as seen in Fig. 2*C*.

### GFET Biosensor Validation.

**Fig. 3.**
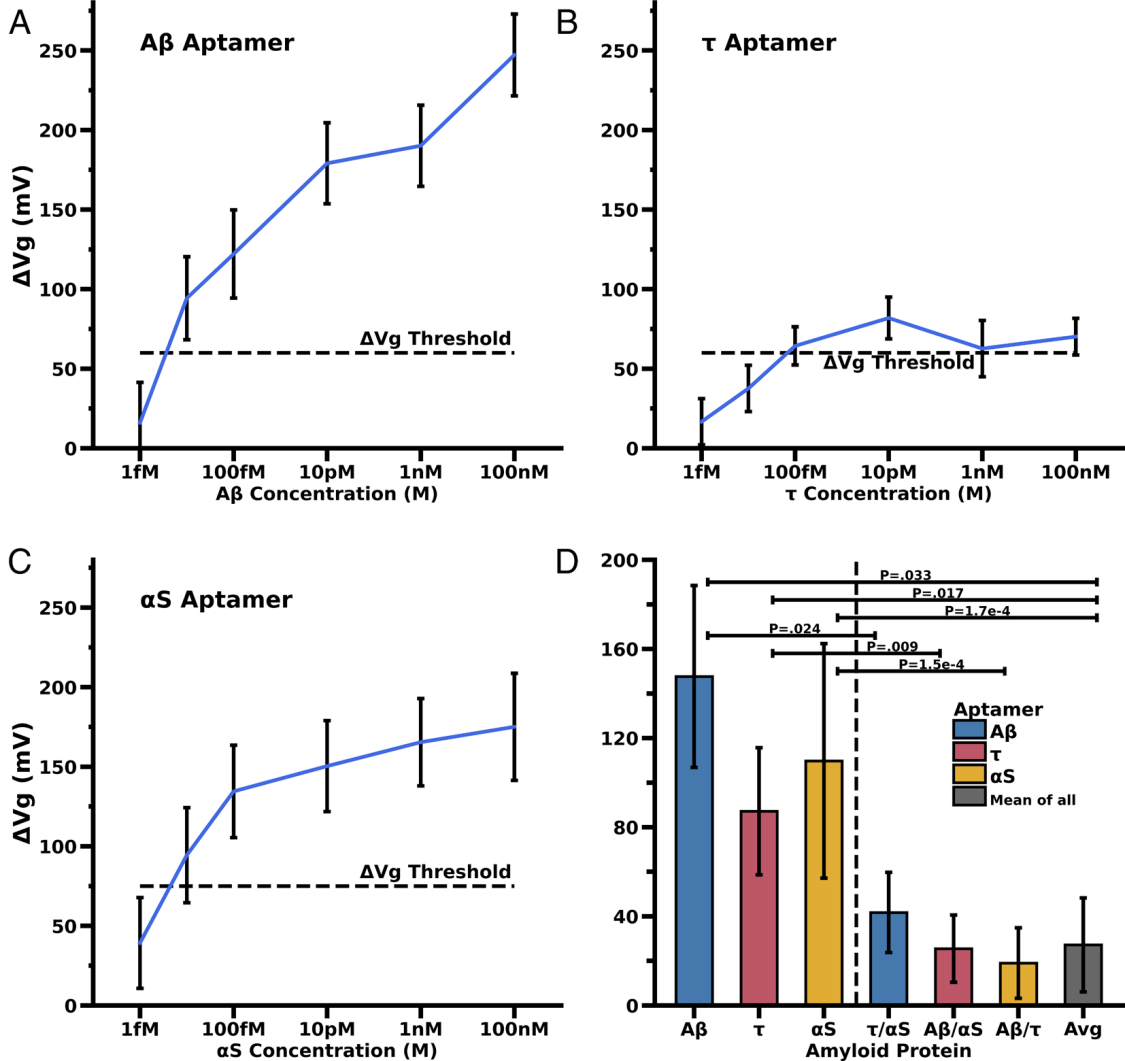
GFET Detection sensitivity and aptamer probe-specificity for Aβ, Tau, and αS proteins. (*A–C*) The plots *A*, *B*, and *C* show concentration-dependent Dirac shift for Aβ, Tau, and αS, respectively. Each curve is derived from independent experiments of the respective proteins with the same concentrations tested (1 fM, 10 fM, 100 fM, 10 pM, 1 nM, and 100 nM). The dotted black line, the sensing Threshold, indicates an SNR ratio of 3 (3 × the PBS control experiments Dirac shift), 60 mV, 60 mV, and 70 mV for Aβ, Tau, and αS respectively. (*D*) Plot *D* is the summary histograms of experimental results supporting the specificity of aptamer probes for their cognate proteins (Aβ_1–42_, Tau, and αS). GFET response of synthetic Aβ, Tau, and αS peptides to their specific aptamers (blue: Aβ aptamer, red: Tau aptamer, yellow: αS aptamer are represented in the first three left bars). The next three bars show the significantly lower, nonspecific response for amyloids tested against their nonspecific aptamer. The last bar from the left (gray bar) indicates an average of the results of all three aptamers tested with their nonspecific cognate proteins. The *x*-axis denotes the analyte protein tested using color-coded aptamers. The positive controls had the correct protein added to the sample (bars to the left of the vertical dotted line). The negative controls are an average of both other proteins added to the incorrect aptamer chip (bars to the right of the vertical dotted line). The result labeled as Avg is an average of each of the negative control nonspecific protein experiments. We illustrate significant *P*-values between the cross-protein controls and correct protein–aptamer Dirac shift results.

To validate the aptamer-GFET biosensor as a quantitative and precise diagnostic tool, we first focused on determining lower limits of detection for each neurodegenerative disease-associated biomarker (Aβ, Tau, αS) by measuring Dirac shifts after the application of various concentrations of these amyloids suspended in 0.1x PBS buffer (Fig. 3). There is a precipitous drop in signal at concentrations <10 fM indicating a possibility to detect concentrations of a biomarker in the femtomolar range but a significant reduction in signal below the 10–100 fM concentration range. Thus, the Aβ and αS aptamer-GFET biosensor can detect proteins at concentrations with the lower LoD of 10 fM (Fig. 3 *A* and *B*, respectively). Tau protein, likewise, can be detected at concentrations approaching 100 fM (Fig. 3*C*).

### Aptamer-specificity for Aβ, Tau, and αS by Cross-control Experiments.

The efficacy of any sensor is reliant on the ability to detect a specific protein or analyte of interest without noise or interference from other nontarget molecules in each sample. One simple experiment to verify this was to test how our sensor, when functionalized with a specific aptamer, will react with other proteins associated with neurodegenerative diseases. We used the aptamers discussed above and the synthetic proteins at a set concentration of 50 nM. We then proceeded to measure the Dirac voltage shifts for each of the combinations and averaged the results of the nonspecific, nontarget protein on each aptamer GFET combination. The results indicate there was not a statistically significant change in signal from adding nontarget proteins to the sample and conclude that our GFET aptamers are specific to the target protein and not to nonspecific adsorption at the surface of the graphene layer (Fig. 3*D*). Fig. 3*D* shows that for a specific aptamer, there is a Dirac shift of ~20 to 30 mV difference between the specific protein and a nonspecific protein. We show significant specificity of the Aβ aptamer to Aβ_42_ but less significant response to Aβ_40_ and no specificity (cross-reactivity) to a nonspecific viral protein in *SI Appendix*, Fig. S4.

### Detection Threshold of GFET Sensors for Aβ, Tau, and αS.

**Fig. 4.**
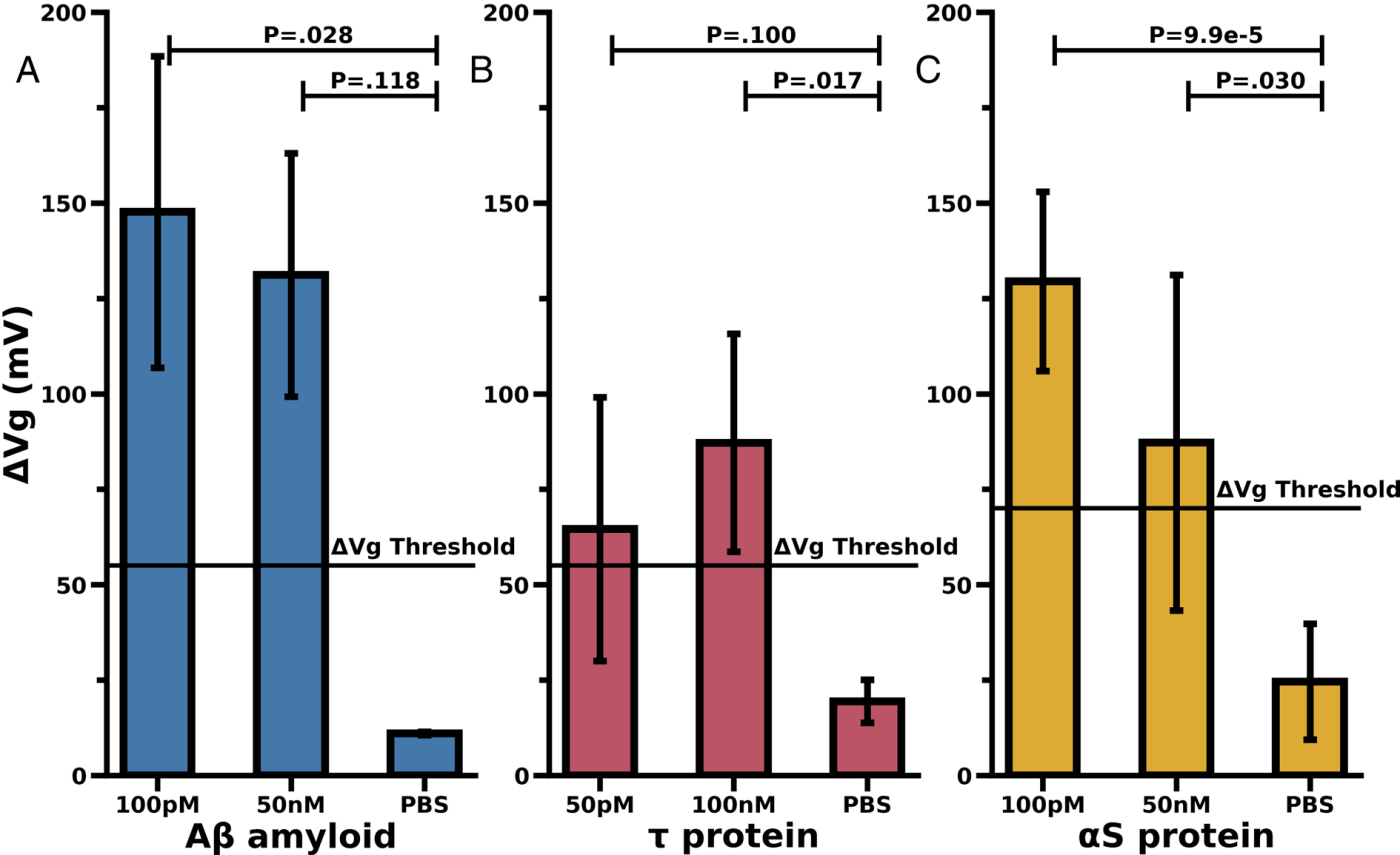
Detection threshold for synthetic Aβ, Tau, and αS protein using their specific aptamer probes. (*A*) Aβ_1–42_ at varying concentrations as well as PBS alone (control) were tested using Aβ aptamer functionalized GFET. (*B*) Tau protein at varying concentrations as well as PBS alone (control) were tested using Tau aptamer. (*C*) αS protein at varying concentrations as well as PBS alone (control) were tested using αS aptamer. Each plot illustrates the significant *P*-values between the synthetic protein and PBS buffer control experiments. The Dirac voltage shift threshold line is shown as a reference for a theoretical cutoff signifying positive from negative results (3 × a PBS control results, see Fig. 3). In the case of Aβ, where higher soluble Aβ_1–42_ levels correlate more with normal cognition rather than an AD state, the greater Dirac shift will relate to less probable AD diagnosis.

The second set of experiments were to measure the sensitivity of the biosensor. We used synthetically derived proteins in a controlled PBS (phosphate-buffered saline) buffer solution to define the detection threshold. We started with a wide range of concentrations of Aβ, Tau, and αS and settled with 1 pM, 100 pM, and 50 nM as a reasonable range of concentrations with respect to the K_D_ (Dissociation constant) values of each aptamer (Fig. 4*A*).

We performed experiments in a similar range of concentrations of 100 nM to 50 pM for the Tau aptamer. We show a significant signal at 100 nM that is higher than the negative control test with pure PBS without any protein (Fig. 4*B*). The last protein we detected was αS at a similar range of protein concentrations (100 pM and 50 nM) in a controlled PBS buffer. We observed a reduced signal at the specified concentrations which, however, is differentiable from the control tests (Fig. 4*C*).

### Detection of Brain-derived Aβ, Tau, and αS Proteins.

**Fig. 5.**
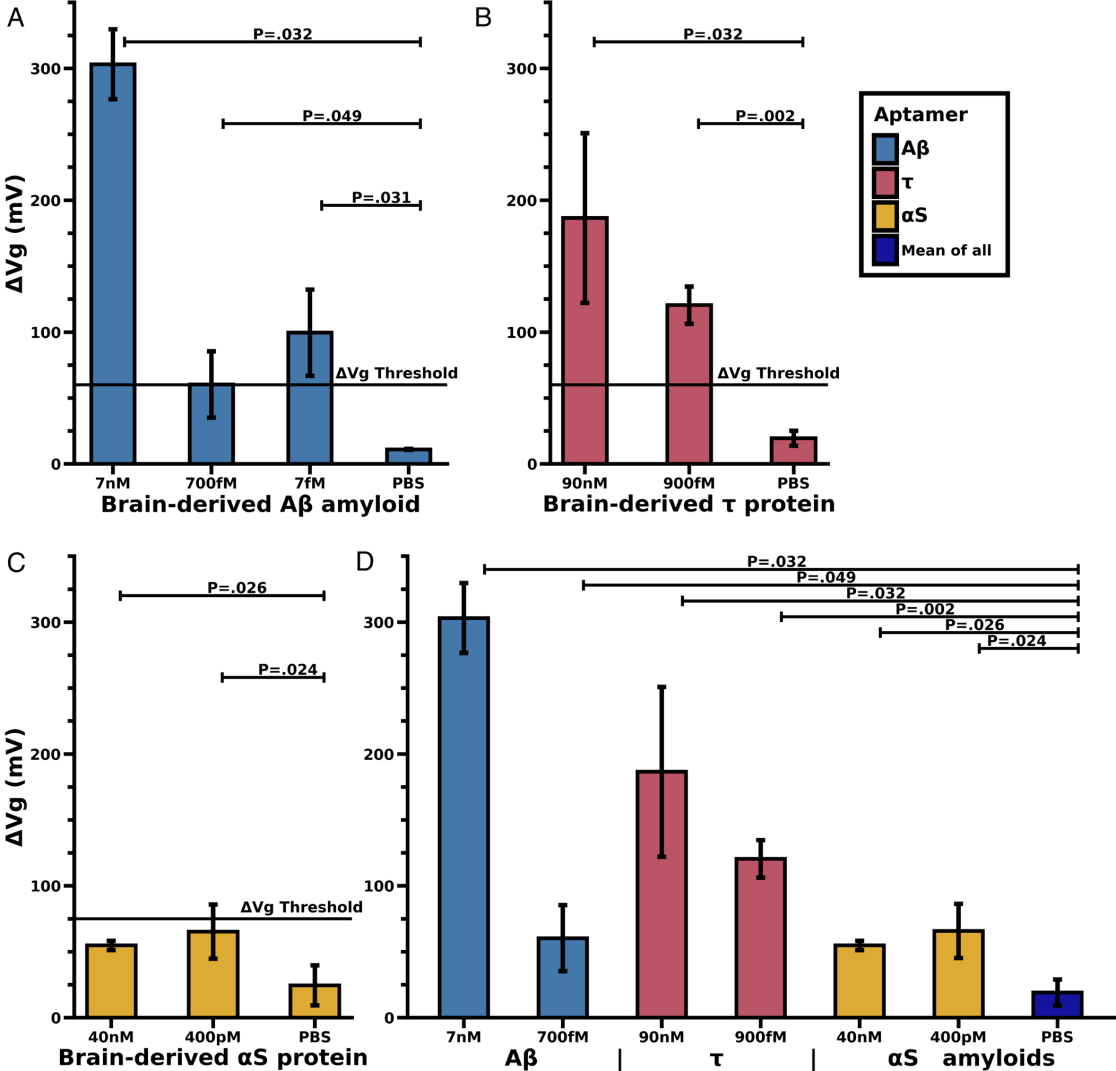
Detection threshold and specificity of AD patients autopsied brain-derived Aβ, Tau, and αS proteins. (*A*) Results of our biosensor against brain-derived Aβ_1–42_ proteins isolated from a diagnosed individual with AD at varying concentrations. (*B* and *C*) Similar experimental results with brain-derived tau protein and αS protein. (*D*) A comparison of the different brain-derived proteins against each other. The significance (*P*-value) of each result is shown with respect to a PBS buffer control. The color of the bars is associated with the aptamer used on the GFET chip (blue Aβ, red tau, yellow αS, gray is an average of all pbs results across aptamers). The solid ΔVg Threshold line is the detection threshold for the Dirac shift in positive samples (3 × a PBS control experiment, see Fig. 3).

Our goal is to detect/diagnose physiological Aβ, tau, and αS from saliva, urine, and other biofluids. Here, we tested our biosensor on brain-derived Aβ, tau, and αS oligomers at various sample dilutions on these amyloids. Brain-derived Aβ shows a dose-dependent response. The lower limit appears to be below the physiological level at a concentration of ~10 fM, which was significantly resolvable w.r.t. to PBS control. The Aβ-aptamer appears to have higher sensitivity than the other aptamers. Tau protein-specific ssDNA aptamer can detect a relatively lower conc of brain-derived Tau (10–100 fM). αS protein-specific ssDNA aptamer was able to detect as low as a 10–100 nM concentration of brain-derived αS (Fig. 5).

The clinically significant levels of Aβ_1–42_ in CSF from AD patients are 614 pg/mL (150 pM) and 864 pg/mL (225 pM) in healthy adults (25). We calculated a detection limit based upon a Dirac shift greater than 3 × SNR (3 × the Dirac shift of PBS control experiment on specific aptamer) (Fig. 3). The GFET sensor’s detection limit of 10 fM for Aβ indicates that our sensor is capable of detecting even a lower Aβ concentration present in later-stage AD patients (Fig. 3*A*). With a detection limit of 1–10 pM for Tau protein (Fig. 3*B*), our GFET sensor can detect Tau in both healthy individuals (300 pg/mL or 5.5 pM) as well as in unhealthy patients (600 pg/mL or 11 pM) (26). With a detection limit of 10–100 fM for synthetic αS (Fig. 3*C*), our GFET sensors can detect αS in patients diagnosed with PD since these patients have higher levels of blood-plasma α-synuclein (3 pg/mL or 200 fM) as compared to healthy control patients (20 fg/mL or 1.33 fM) (27).

## Discussion

The world is continuing to face an increasingly aging population, which is exacerbated by declining birth rates and increased life expectancy. This brings to the forefront of modern medicine the need to better understand, prevent, and treat elderly patients who are at risk of developing AD, PD, and other neurodegenerative diseases. In addition to age-related risk factors, traumatic brain injury (TBI) is being studied as a potential risk factor for the development of neurodegenerative disease such as AD or encephalopathy (28). Studies have shown an increasing link between the development of Tau fibrils and Aβ amyloid plaques with a common upstream pathology (29). Though there is yet to be a successful cure, there has been continual effort to design therapies that treat Aβ generation and degradation pathways as a means of alleviating the symptoms and progression of AD (30). In this study, we have examined the ability of our GFET biosensor, which is able to detect as few as seven Sars-CoV2 viruses [and 100 spike and nucleoproteins per 10 μL sample (18, 31, 32)], for early, simple, at-home, and POC detection of Aβ, Tau, and αS, biomarkers for AD, PD, and neurodegenerative diseases.

To determine the lower LoD, we first used synthetic Aβ, Tau, and αS proteins. As summarized in Fig. 3, our GFET biosensor can detect 10 fM Aβ, 1–10 pM Tau protein, and 10–100 fM αS at a statistically significant level versus control. These detection limits are within the concentration ranges for Aβ, Tau, and αS proteins in normal as well as diseased patients (25–27).

As Aβ_1–42_ can be considered as an intrinsically disordered protein (IDP), we wanted to confirm the affinity of the aptamer used for Aβ_1–42_ against an amino acid scrambled variant of Aβ_1–42_. As an additional control, the results indicate that our GFET sensor can significantly distinguish Aβ_1–42_ from its scrambled variant supporting our assertion that the Aβ aptamer we have used in our study is specific to the 3D conformation associated with Aβ_1–42_ and is not affected by the nonspecific binding of proteins with similar net charge or size (*SI Appendix*, Fig. S3).

A comparison of the different proteins and aptamer combinations indicates that the most effective apta-sensor is likely the Aβ and αS aptamer and protein combination, respectively (Figs. 3 and 4). It appears that the Tau protein is less easily detected using our combined aptamer GFET biosensor than the other neurodegenerative proteins. This could be in part due to the molecular charge of these amyloids—Aβ and αS both have negative net charges, whereas Tau protein has a largely positive net charge (33, 34). The negative charge of the protein helps to increase the sensitivity of our sensors. The GFET biosensors we have developed are P-doped, as shown by the positive gate voltage at the Dirac point indicating holes to be the majority charge carriers. As such, the negative molecules will trigger an even more positive shift and a reduced shift in the case of the positive Tau protein (35). In addition, the reduced signal for tau using the Tau-aptamer could results from two different factors. First, as a steric effect—Tau is nearly an order of magnitude larger than Aβ and αS and thus inducing a greater steric interference between unbound aptamers in close proximity to a Tau-bound aptamer. Second, the relatively low shift at 3 × SNR in the Tau aptamer synthetic case (Fig. 3*B*) could be partly due to the Tau aptamer having been developed against phosphorylated Tau which is more likely to be present in brain-derived Tau samples.

Our overarching goal was to develop a simple sensor for detecting Aβ, Tau, αS, and other neurodegenerative proteins in the body. Our natural next step was to conduct experiments with brain-derived proteins to assess whether the system behaved similarly for in vivo derived proteins. Fig. 4 indicates that the aptamer-GFET biosensor can detect a significant signal at similar concentrations and dilutions as the synthetic proteins. Fig. 5 summarizes our finding that the autopsied brain-derived neurodegenerative proteins (Aβ, Tau, and αS) bind with significant specificity to the aptamer biosensor and not to other nontarget proteins. The signal produced from brain-derived Tau appears to be larger compared to the signal described in the concentration plot for the synthetic Tau. The increase in signal could be partly because the aptamer used in the work was selected against phosphorylated Tau, the form present in the AD brain-derived samples (*SI Appendix*, Table S1). This makes the biosensor a strong candidate for diagnostics as it will limit nontarget binding and false-positive results and will also allow for greater lower limits of detection in human samples (Fig. 3). We are continuing our work with experiments to confirm our biosensor’s capacity to detect neurodegenerative proteins in complex biological samples such as CSF and saliva.

Though our experiments show promising results, we acknowledge certain limitations. Detecting brain-derived amyloid proteins directly via bodily fluids presents additional challenges in sample preparation to limit nontarget molecular interactions with the sensor, among other issues. We are currently undertaking these studies with CSF and saliva focusing on measuring relative change in concentrations of Aβ_1–42_, Tau441, and αS over time as a means of monitoring disease progression. It is posited that the ratio of relative concentrations of Aβ_1–42_ and Aβ_1–40_ may be a more feasible indicator of AD pathogenesis than Aβ_1–42_ concentration alone (5, 10). A multifaceted, comparative study to identify various amyloid oligomer isoforms for diagnosis of early and late-stage AD and PD as well as to understand the role of regional, genetic, and population diversity would require more specific aptamers for each amyloid isoform. The aptamer-functionalized GFET biosensor platform and methods described in the present work provide a direct and viable pathway to achieve the goal of effective diagnosis of neurodegenerative diseases.

## Materials and Methods

### Brain-Derived Tau 441 and αS.

Tau441 and αS were supplied and characterized by Rakez Kayed’s group at UTMB (36, 37). The preparation methodology is as follows.

### Brain Homogenization.

Postmortem brain tissues were acquired from Oregon Health and Science University, the Institute for Brain Aging and Dementia (University of California–Irvine, Irvine, CA), and the Brain Resource Center at Johns Hopkins. Neuropathological assessment followed the consensus criteria established by the National Institute on Aging/Reagan Institute. Postmortem brain tissue of AD patients was homogenized in PBS containing a protease inhibitor cocktail (Roche; 11836145001) using a brain-to-PBS dilution ratio of 1:3 (w/v). The samples were subsequently subjected to centrifugation at 10,000 rpm for 10 min at 4 °C. The resulting supernatants were aliquoted, rapidly frozen, and preserved at −80 °C until further use.

### Immunoprecipitation of Toxic Tau.

Immunoprecipitation of toxic tau was performed as described previously (36–40). Briefly, tosyl-activated magnetic Dynabeads (Dynal Biotech, Lafayette Hill, PA) were coated with 20 μg of T18 antibody (1.0 mg/mL) diluted in 0.1 M borate, pH 9.5, overnight at 37 °C. The beads were washed and exposed with PBS-soluble AD postmortem brain homogenate. The homogenate and bead mixture were incubated at room temperature for 1 h. The beads were washed three times with PBS and eluted using 0.1 M glycine, pH 2.8. The pH was then neutralized using 1 M Tris base. The samples were then quantified using bicinchoninic acid protein assay and stored at −80 °C until further use.

### Purification of Recombinant Tau and Amplification of Brain-derived Tau Aggregates.

The human tau-441 isoform (2N4R) was expressed as a recombinant in *Escherichia coli* BL21 (DE3) cells and purified as described previously (41, 42). The monomer was seeded with brain-derived tau at a ratio of 1:100 (w/w) with a rotation of 48 h at 37 °C (40, 43–45). The samples were characterized using SDS-PAGE followed by western blotting and AFM. The samples were then flash-frozen until further use. αS were also expressed in *E. coli* BL21(DE3) cells as described above (42). The purified tau proteins when characterized with the published methods described previously (36, 38, 40) show both monomeric and dimeric forms.

### Immunoprecipitation of αS.

αS oligomers were immunoprecipitated using F8H7 (a-synuclein) antibodies. IP was carried out following the manufacturer’s recommendations (Thermo Scientific Cat No. 23600). Brain tissue of PD patients was homogenized in PBS with protease inhibitor cocktail (Cat.11836145001, Roche Diagnostic). The samples were centrifuged at 10,000 rpm for 10 min at 4 °C. The αS brain-derived samples when characterized by gel electrophoresis and silver staining show that post immunoprecipitation, the oligomers are mainly monomers, dimers, and trimers (*SI Appendix*, Fig. S5).

### Graphene Field-effect Transistor Fabrication and Characterization.

The fabrication process was slightly modified from previously published GFET work (18). The graphene was synthesized by low-pressure chemical vapor deposition (LPCVD) on 25-µm-thick copper foil (MTI Corp.), then it was spin-coated at 3,000 rpm for 45 s by 120 K molecular weight poly methyl methacrylate (PMMA) for a PMMA-assisted wet transfer process. Oxygen plasma etching was applied to remove the graphene on the backside of the copper foil (18). Ferric chloride solution was used to etch copper foil and subsequently rinsed with DI water. The large-sized PMMA/graphene film was transferred on a 4-inch SiO_2_ /Si substrate with 100-nm-thick Au/Cr electrodes. For 1 h, the PMMA was dissolved via acetone treatment, which was subsequently, followed by an application of isopropyl alcohol (IPA) rinse and nitrogen blow-dried. To protect the graphene channels and define a 500-µm graphene channel length, photolithographic micropatterning methods with PMGI photoresist were utilized. Excess graphene was removed via oxygen plasma etching (18). Followed by the removal of photoresist, the surface of graphene was further annealed at 200 °C for 2 h under forming gas atmosphere to anneal impurities (18). Raman spectroscopies were performed on 96 consecutive points on the given brightfield image with 20-μm pitch, which allowed us to map the intensities (*SI Appendix*, Fig. S2 *A* and *B*) as well as resistance measurements confirmed graphene monolayer quality of the GFET chips.

After dicing the patterned wafer, the GFET chips were glued to a PCB board/chip carrier and the gate, source, and drain terminals were wire bonded to the contact pads. The Au/Cr electric pads and wire bonds were shielded from direct contact with the electrolyte solution with silicone paste, and a well (3–5 mm internal diameter), made of silicone tubing, was glued onto the chip to serve as a reservoir during derivatization and sample incubation (18). This process was automated at the SIMIT facility of Tie Li and Jianlong Zhao. Additional characterization data were obtained using Raman spectroscopy and AFM, see *SI Appendix*.

The remaining methods that detail chip functionalization, aptamer selection, experimental procedure, reagent preparation, and more are also present in *SI Appendix*.

## Supplementary Material

Appendix 01 (PDF)

## Data Availability

All study data are included in the article and/or *SI Appendix*.
