# Supplementary material for: In pursuit of degenerative brain disease diagnosis: Dementia biomarkers detected by DNA aptamer-attached portable graphene biosensor
Source: Proc Natl Acad Sci U S A. 2023 Nov 13;120(47):e2311565120. doi: 10.1073/pnas.2311565120 (PMC10666025; doi:10.1073/pnas.2311565120)
Supplement: Supplementary file 1 — Appendix 01 (PDF) [file pnas.2311565120.sapp.pdf]

Supporting Information for

In pursuit of degenerative brain disease diagnosis: dementia biomarkers detected by DNA aptamer-attached portable graphene biosensor

Tyler Andrew Bodily[1]†, Anirudh Ramanathan[1]†, Shanhong Wei[2,3]†, Abhijith Karkisaval[4]†, Nemil Bhatt[5], Cynthia Jerez[5], Md Anzarul Haque[5], Armando Ramil[1], Prachi Heda[1], Yi Wang[2], Sanjeev Kumar[6], Mikayla Leite[1], Tie Li[2]*, Jianlong Zhao[2]*, Ratnesh Lal[1,4,7]*

[1]Department of Bioengineering, University of California San Diego, [2]Shanghai Institute of Microsystem and Information Technology, CAS, Shanghai, China, [3]University of Chinese Academy of Sciences (UCAS), Beijing, China, [4]Department of Mechanical and Aerospace Engineering, University of California San Diego, [5]Mitchell Center for Neurodegenerative Disorders, Department of Neurology, University of Texas Medical Branch, Galveston, TX, [6]University of Illinois Urbana-Champaign (UIUC), [7]Materials Science and Engineering Program, University of California San Diego

Tie Li, Jianlong Zhao, Ratnesh Lal

Email: tli@mail.sim.ac.cn, jlzhao@mail.sim.ac.cn, rlal@eng.ucsd.edu

This PDF file includes:

Supporting text

Figures S1 to S5

Tables S1

SI References

More Materials and Methods

Reagents

Synthetic Aβ_{1-42}, Tau441, and αS were purchased from [Anaspec] Standard desalted quality 3'amino functionalized oligonucleotide aptamer probes for Aβ_{1-42}, Tau441, and αS were used. The aptamers were obtained from IDT [Integrated DNA Technologies]. The nucleotide sequences are included in the supplementary material (see Table S1). Molecular biology grade 1x PBS [Gibco], $MgCl_2$ [Invitrogen], DMF [Sigma-Aldrich], Isopropyl Alcohol [Acros], Acetone [Fischer], and Ethanol [Decon Labs] were used throughout the study. Analytical-grade PBASE [Invitrogen] and ethanolamine [Alfa Aesar] were used without further processing.

Brain Derived Aβ_{1-42}

We obtained brain-derived samples of Aβ_{1-42} from Dr. Jorge Ghiso's lab at NYU. All samples were collected with informed consent and according to institutional regulations set. Briefly, postmortem brain tissue from AD patients were homogenized in PBS buffer with protease inhibitors. Purified Aβ_{1-42} was extracted from the PBS fraction via immunoprecipitation (using antibodies 4G8 and 6E10). The purified Aβ samples were then aliquoted and stored in -80C until further use. Published work shows that the purified brain-derived Aβ samples are monomeric [1].

GFET Sample Preparation

Our experiments were performed in a BSL2 facility. The synthetic proteins which are in lyophilized form were diluted in 0.1 M sodium phosphate buffer and stored in 5 µL aliquots capable of serial dilution of the sample to the desired concentrations prior to testing. Brain-derived amyloid protein stock was received in buffer form with a concentration of 0.3-0.5 mg/mL and stored in 5 µL aliquots. The serial dilutions to the required concentrations were made in 0.1x PBS with 0.5 mM $MgCl_2$ which acts as our standard buffer throughout this manuscript. These monomer dilutions would be prepared fresh (stored on ice) and used immediately or within 3-6 hours in order to prevent oligomerization.

Aptamer Selection and Preparation

Based on an extensive literature review, we shortlisted an aptamer for every protein based on the lowest K_d. Aβ, tau, and αS aptamers were selected for this study (Table S1). The Aβ aptamer was selected against $Aβ_{1-42}$ monomers and the αS aptamer was specifically selected to bind to monomers. It is unclear whether the SELEX process of the Tau aptamer was done with monomeric or oligomeric Tau, but the aptamer was specifically designed for phosphorylated-Tau (pTau). The aptamer sequences had an amino modification on the 3' end and manufactured such that they would come pre-diluted in IDTE buffer at pH 8.0 with a 100 μM concentration. This would be further dissolved using 1x PBS and 0.5 mM $MgCl_2$ to 1 μM. This aptamer would now be stored at -20 ℃ until further use. The specificity of the biosensors was tested with scrambled amino acid sequence amyloid-beta protein (Figure S3). And finally, the cross-reactivity of each aptamer with their non-specific amyloid proteins (i.e., aptamer for Aβ tested for τ and αS, aptamer for τ tested for Aβ and αS, and aptamer for αS tested for Aβ and τ (Figure 3D) were also tested.

Our Aβ aptamer was chosen as it is specific to the $Aβ_{1-42}$ strand monomer (see Table S1). We tested the affinity of our aptamer to a specific 3D structure of Aβ and compared the performance to a scrambled amino acid variant $Aβ_{1-42}$ (Figure S3). The secondary and tertiary structures of the scrambled Aβ were different enough to indicate that our aptamer/sensor does not simply rely on electrostatic interactions but is also reliant on the secondary/tertiary structure of the target $Aβ_{1-42}$ [2]. The results in Figure S3 indicate a significantly different sensor response compared to pure PBS controls and scrambled and non-scrambled sequences of Aβ (p-value < 0.05).

Chip Functionalization

In order for the chips to specifically bind and target the proteins of interest we needed to modify the graphene surface with the aptamers specifically developed for binding said target proteins. This was achieved by first applying a layer of PBASE (1-Pyrenebutyric acid N-hydroxysuccinimide ester) to the graphene surface. The pyrene ring will bind to the graphene

surface by pi-bond interactions, known as pi-stacking. NHS modifications of the PBASE molecule bind to the amine group at the 3' end of the amine-modified ssDNA and allow the aptamer to fold freely on its own and bind to the target protein. The free NHS groups are passivated by adding ethanolamine after the aptamer addition step. The amine group binds to the NHS ester and eliminates the potential for reactivity with free-floating amine groups on non-target proteins and molecules.

Experimental Procedure

Once the chips are functionalized, the buffer is added to the chip well and allowed to sit in moist conditions for a day to avoid potentially significant drift in the Dirac voltage and bring some stability [1]. After 24 hours, the well is washed with a buffer to remove any salt deposits. Finally, 10 μL of the buffer is added to the chip well and measured using the PIVOT device [3]. This PBS Dirac point acts as our baseline measurement. Anywhere between 3 to 4 such baseline measurements in 10-minute intervals are taken to reach a relatively consistent characteristic Dirac voltage. Following this, 10 μL of the required sample is added and incubated for 10 minutes. We then measure this new addition on the PIVOT device, where 3 to 4 consecutive measurements are taken in 10-minute intervals. This would act as the sample Dirac voltage. The difference between the final baseline measurement and sample Dirac voltage would constitute the characteristic Dirac shift for a given chip, and this was repeated on many such chips. Every chip has its own characteristic measurements and will be different from the others therefore, the use of chip-specific baseline calculations is critical. The PIVOT device can be connected to a computer remotely for data transmission which would enable clinicians to easily access patient data.

Quality control metrics

The GFET biosensors for this experiment were procured from the Shanghai Institute of Microsystem and Information Technology (SIMIT). The sensitivity of graphene as a biosensor template requires a uniform monolayer of graphene for optimal functionality. GFET mass production introduced challenges with consistent data and results from the variation of the

surface layer. We started by controlling for the drain-source resistance and limited our studies to biosensors that were within the range of 1-10 kΩ. From there we developed an algorithmic approach to identify the non-functioning chips automatically. They are easily distinguishable by their characteristic low signal-to-noise ratio and flattened I-V curve shape, as opposed to the characteristic graphene U-shaped I_{DS} vs V_{GS} curve (ambipolar I-V curve). We first fit a hyperbolic curve to the I_{DS}-V_{GS} plot of the graphene voltage sweep. We did this for 100 good and bad data sets and found the relative distribution of parameter values (a,b,c,d) (see equation 1). A threshold value was set to 3 times the standard deviation beyond the minimum and maximum values for each of the parameters. This fit was then applied to each new dataset and a score was calculated based on how many of these parameters were outside the established range of good data parameter values. This same methodology was also used for a basic parabolic graph fit and the same parameter range and curve scoring method was used for determining the likelihood of a poor graphene chip. Additional scores were calculated from the peak-to-peak variation by finding the difference from the low-pass filtered data and a test fitting straight lines to both linear regions on either side of the Dirac point. The results of these quality control experiments are summarized in the supplementary information (Figure S1).

Eq 1: $$\frac{(x-c)^2}{a^2} + \frac{(y-d)^2}{b^2} = 1$$

Statistical Analysis

We excluded certain datasets depending on the validity of the data generated from the GFET biosensor chips. We took multiple baseline measurements, then excluded those results that showed a greater than 8% variation in Dirac point among each I-V sweep to eliminate those chips that have significant drift in a control buffer solution and improve the sensitivity of our biosensor results. We conducted additional quality checks by measuring the drift in consecutive sweeps on a single chip. If a baseline buffer sample showed a significant coefficient of variation greater than 0.08, we also excluded those chip results as this could be indicative of a faulty GFET chip and surface impurities. We calculated the p values using a standard heteroscedastic two-tailed student t-test for all results presented. The standard deviation of multiple Dirac sweeps would indicate the error in the Dirac point for a given chip. The average error of multiple chips would be the standard deviation of the Dirac points from the multiple chips. The error bars in each graph are indicative of the average plus the standard deviation across multiple chips (3-5). The LOD was calculated as the concentration at which signal reaches 3x SNR (signal to noise ratio). The baseline signal was determined as the Dirac shift of 4-5 chips averaged together in PBS buffer (20 mV Aβ, 20 mV Tau, 25 mV αS).

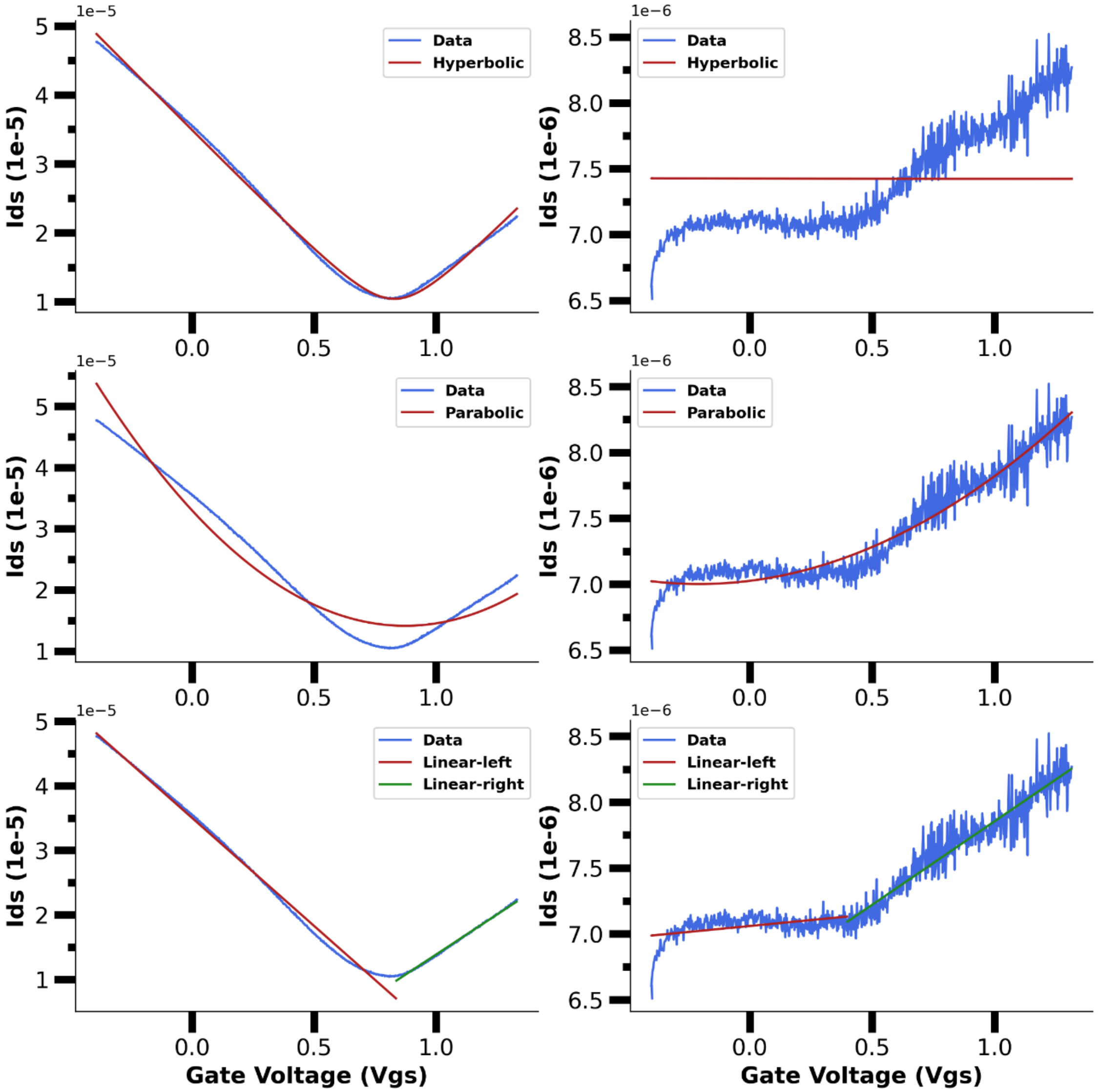

Figure S1. A display of the examples of three of the metrics used in the quality control screening developed for optimizing the GFET chip functionality. The first column is a good chip, and the second column is a bad chip. The top graphs are from a hyperbolic fit with the raw data. The middle graphs are a parabolic fit and the bottom graphs are a linear fit to either side of the linear regime of the Dirac curve.

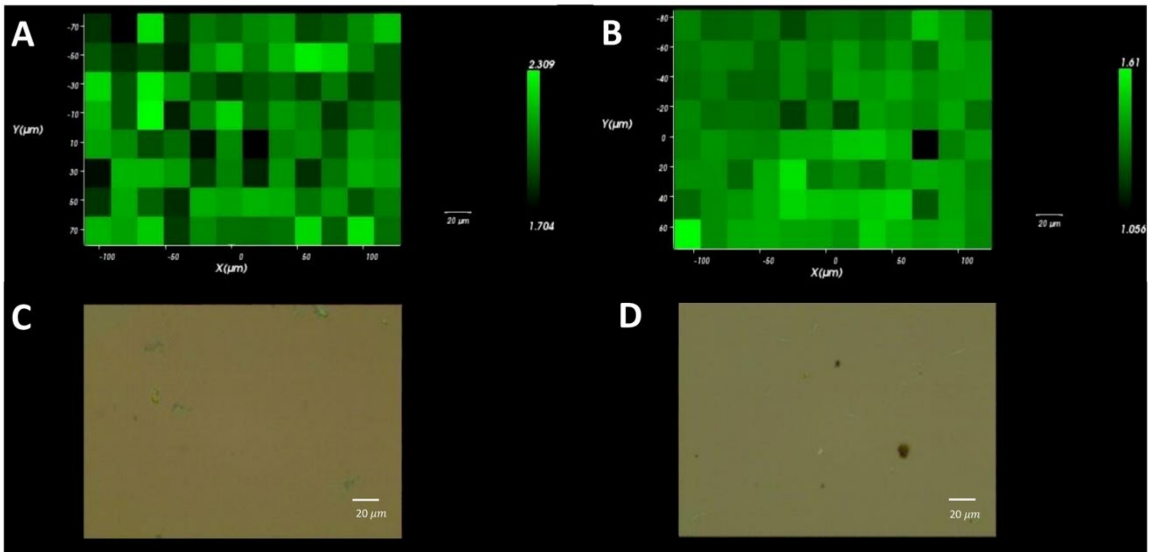

Figure S2. A. Raman map of unmodified functionalized FET sensor of a surface area of 150 x 250 μm^2 with spectroscopies taken at a pitch of 20 μm, with a total of 96 points. B. Raman map of PBASE functionalized FET sensor of a surface area of 150 x 250 μm^2 (same scale and periodicity as A) C. Brightfield image (20x magnification) of the unmodified graphene surface of the FET sensor (with the same surface area as A). D. Brightfield image of PBASE functionalized GFET sensor (with the same surface area as B).

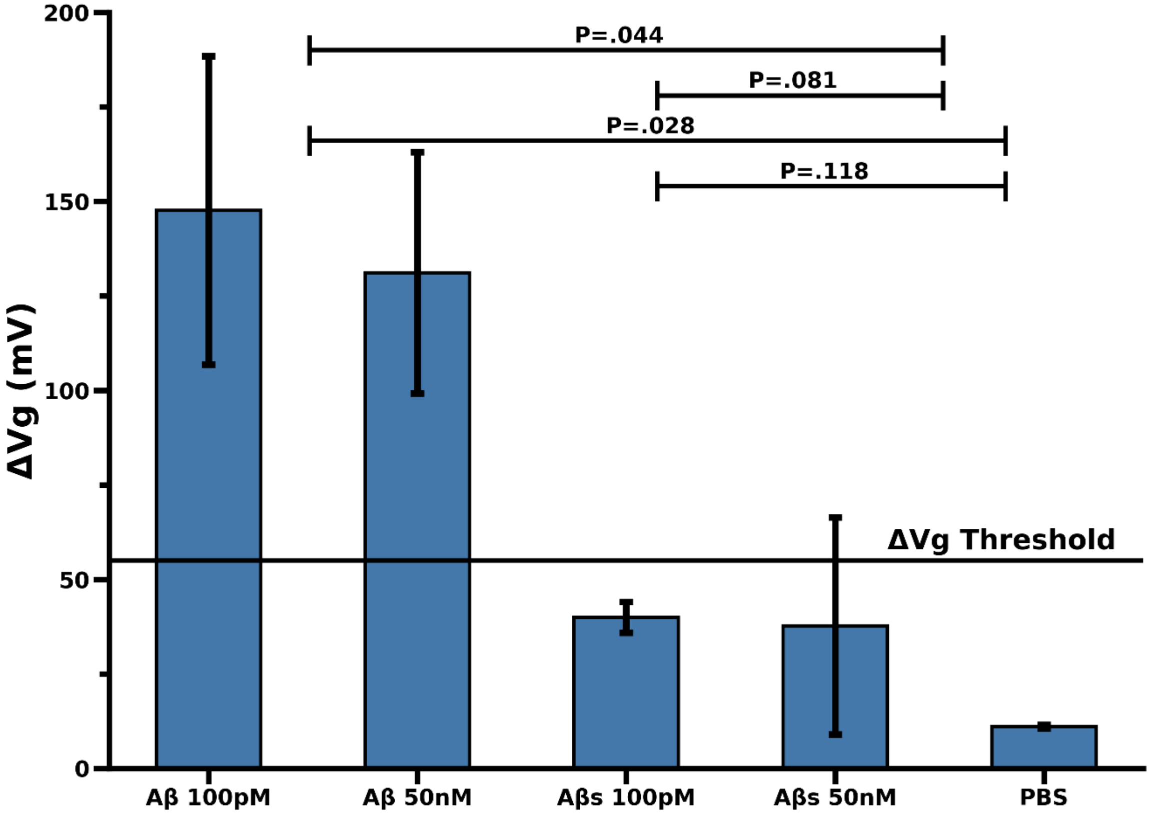

Figure S3. The figure depicts experiments done on amyloid-beta aptamer functionalized GFET biosensors at two concentrations (100pM and 50nM). We compare Dirac shifts between the biologically correct amino-acid sequence synthetic Aβ and a randomly scrambled amino-acid sequence Aβs. The results indicate the amino-acid sequence specific shift in the Dirac point significantly above the detection threshold (Figure 3) when using the Aβ aptamer-functionalized GFET biosensors. These experiments highlight the specificity of our aptamer biosensor to the specific amyloid-beta sequence.

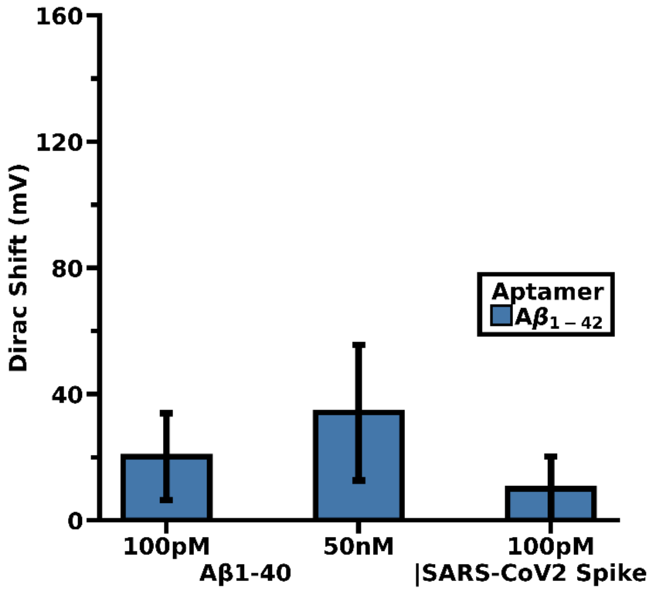

Figure S4. The specificity of Aβ aptamer for the detection of Aβ$_{1\text{-}42}$. Aβ$_{1\text{-}40}$, when tested with Aβ$_{1\text{-}42}$ aptamer-functionalized GFET biosensors showed significantly non-specific response compared to testing Aβ$_{1\text{-}42}$ proteins (Figures 3-4). Similarly, a non-amyloid protein (SARS-CoV2 spike protein) when tested using the Aβ aptamer shows significantly non-specific response. The reduced response to Aβ$_{1\text{-}40}$ compared to that of Aβ$_{1\text{-}42}$ indicates that with an aptamer specific to Aβ$_{1\text{-}40}$ the quantification of the ratio of Aβ$_{40}$/Aβ$_{42}$ for diagnostic purposes could be possible. Additional controls with non-specific proteins from SARS-CoV2 are also shown against the Aβ$_{1\text{-}42}$ aptamer. Previous work highlights the functionality of SARS-CoV2 Spike protein detection using aptamers specific to the Spike protein with the GFET biosensors [4].

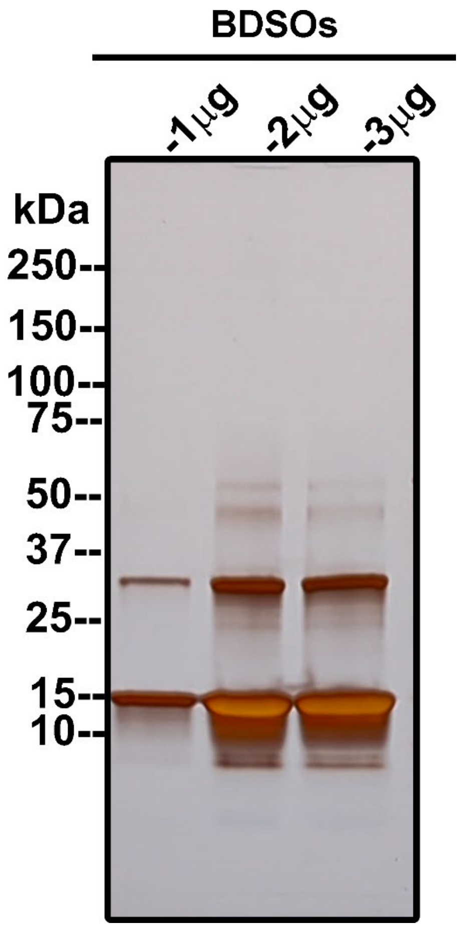

Figure S5: Alpha-synuclein oligomers derived from the brain (BDSOs) were loaded onto an SDS-PAGE gel at 1-3 µg. Subsequently, the gel was silver stained using the Pierce Silver Stain Kit (ThermoFisher, Cat# 24612) according to the manufacturer's instructions. The gel revealed the presence of dimers and trimers (~30kDa and ~45kDa, respectively), in addition to monomeric species (~15kDa).

Table S1. The table above identifies the different aptamers we used in the detection of the three neurodegenerative proteins via GFET functionalization. The relative K_D values and sequences are also included.

**Analyte (Aptamer name)**	**kD (nM)**	**Sequence (5'-3')**
Amyloid-β (Aβ7-92-1H1) [5]	63.4	CCG GTG GGG GAC CAG TAC AAA AGT GGG TAG GGC GGG TTG GAA AA
α-synuclein (F5R1) [6]	2.4	ATC GAG TGT GTA CGG GGT CCG GTA GGG TGG CGA GGT CTT CCT GTC GTA GCA GGA TCC A
Tau441 (IT2) [7]	5.5	CAG CAC CGT CAA CTG AAT AAG GAC TGC TTA GGA TTG CGA TGA TTC AGG GTG ATG CGA TGG AGA TGT

SI References

1. J. Ghiso, A. Rostagno, Y. Tomidokoro, T. Lashley, M. Bojsen-Moller, H. Braendgaard, G. Plant, J. Holton, R. Lal, T. Revesz, B. Frangione, Genetic Alterations of the *BRI2* gene: Familial British and Danish Dementias, *Brain Pathology,* **16,** 71-79, (2006)
2. R. Sabaté, A. Espargaró, N.S. de Groot, J.J. Valle-Delgado, X. Fernández-Busquets, S. Ventura, The Role of Protein Sequence and Amino Acid Composition in Amyloid Formation: Scrambling and Backward Reading of IAPP Amyloid Fibrils. *J. Mol. Biol.* **404**, 337-352 (2010).
3. N. Miyakawa, A. Shinagawa, Y. Kajiwara, S. Ushiba, T. Ono, Y. Kanai, S. Tani, M. Kimura, K. Matsumoto, Drift Suppression of Solution-Gated Graphene Field-Effect Transistors by Cation Doping for Sensing Platforms. *Sensors* **21**, 7455 (2021).
4. D.K. Ban, T. Bodily, A.G. Karkisaval, Y. Dong, S. Natani, A. Ramanathan, A. Ramil, S. Srivastava, P. Bandaru, G. Glinsky, R. Lal, Rapid self-test of unprocessed viruses of SARS-CoV-2 and its variants in saliva by portable wireless graphene biosensor. *Proc. Natl. Acad. Sci. U.S.A.* **119**, e2206521119 (2022).
5. Y. Zheng, P. Wang, S. Li, X. Geng, L. Zou, M. Jin, Q. Zou, Q. Wang, X. Yang, K. Wang, Development of DNA Aptamer as a β-Amyloid Aggregation Inhibitor, *ACS Appl. Bio Mater.* **3**, 8611-8618, (2020)
6. Y. Zheng, J. Qu, F. Xue, Y. Zhang, B. Yang, Y. Chang, H. Yang, J. Zhang, Novel DNA Aptamers for Parkinson's Disease Treatment Inhibit α-Synuclein Aggregation and Facilitate its Degradation. *Mol. Ther. Nucleic Acids* **11**, 228-242 (2018).
7. I.-T. Teng, X. Li, H.A. Yadikar, Z. Yang, L. Li, Y. Lyu, X. Pan, K.K. Wang, W. Tan, Identification and Characterization of DNA Aptamers Specific for Phosphorylation Epitopes of Tau Protein. *J. Am. Chem. Soc.* **140**, 14314-14323 (2018).
